# A multicenter high-quality data registry for advanced proton therapy approaches: the POWER registry

**DOI:** 10.1186/s12885-024-12059-2

**Published:** 2024-03-12

**Authors:** Daniela Alterio, Maria Giulia Vincini, Stefania Volpe, Luca Bergamaschi, Mattia Zaffaroni, Sara Gandini, Giulia Peruzzotti, Federica Cattani, Cristina Garibaldi, Barbara Alicja Jereczek-Fossa, Roberto Orecchia

**Affiliations:** 1 Division of Radiation Oncology, European Institute of Oncology IRCCS, Milan, Italy; 2 Department of Oncology and Hemato-oncology, University of Milan, Milan, Italy; 3 Molecular and Pharmaco-Epidemiology Unit, Department of Experimental Oncology, European Institute of Oncology IRCCS, Milan, Italy; 4 Clinical Trial Office, European Institute of Oncology IRCCS, Milan, Italy; 5 Medical Physics Unit, IEO, European Institute of Oncology IRCCS, Milan, Italy; 6 Unit of Radiation Research, IEO, European Institute of Oncology IRCCS, Milan, Italy; 7 Scientific Directorate, European Institute of Oncology IRCCS, Milan, Italy

**Keywords:** Proton therapy, Registry, High-quality dataset, Hypofractionation

## Abstract

**Background:**

Paucity and low evidence-level data on proton therapy (PT) represent one of the main issues for the establishment of solid indications in the PT setting. Aim of the present registry, the *POWER* registry, is to provide a tool for systematic, prospective, harmonized, and multidimensional high-quality data collection to promote knowledge in the field of PT with a particular focus on the use of hypofractionation.

**Methods:**

All patients with any type of oncologic disease (benign and malignant disease) eligible for PT at the European Institute of Oncology (IEO), Milan, Italy, will be included in the present registry. Three levels of data collection will be implemented: Level (1) clinical research (patients outcome and toxicity, quality of life, and cost/effectiveness analysis); Level (2) radiological and radiobiological research (radiomic and dosiomic analysis, as well as biological modeling); Level (3) biological and translational research (biological biomarkers and genomic data analysis). Endpoints and outcome measures of hypofractionation schedules will be evaluated in terms of either Treatment Efficacy (tumor response rate, time to progression/percentages of survivors/median survival, clinical, biological, and radiological biomarkers changes, identified as surrogate endpoints of cancer survival/response to treatment) and Toxicity. The study protocol has been approved by the IEO Ethical Committee (IEO 1885). Other than patients treated at IEO, additional PT facilities (equipped with Proteus®ONE or Proteus®PLUS technologies by IBA, Ion Beam Applications, Louvain-la-Neuve, Belgium) are planned to join the registry data collection. Moreover, the registry will be also fully integrated into international PT data collection networks.

## Introduction

External beam radiation therapy (EBRT) can be administered using different fractionation schedules. Hypofractionation (dose/fraction > 2.5 Gy) represents the standard of care for many photon-based treatments both for curative and palliative aims. Compared to the standard fractionation schedule (1.8-2 Gy/fraction), hypofractionation has several advantages, such as higher radiobiological efficacy, shorter overall treatment time, and reduction of patients’ logistic discomfort. As for photon-based radiation therapy (RT), the use of hypofractionation is constantly increasing also in the setting of patients treated with proton therapy (PT) [1].

Several studies have demonstrated the feasibility and efficacy of PT administered by hypofractionation.

Therefore, this schedule could be proposed for different histologies of tumors located in different body sites, such as breast [2], lung [3, 4], prostate [5], liver [6], as well as for reirradiation treatments [7]. Despite this, literature data provides mainly data collected as mono-institutional experience or referred to patients enrolled in clinical trials. The paucity of PT data represents one of the main barrier for the establishment of solid indications in the PT setting. As a matter of fact, in addition to the relative scarcity of data, most of them are collected in a non-standardized or fragmented manner.

Therefore, the aim of the present registry, the *POWER* registry, is to prospectively and systematically collect data on patients treated with PT in order to add on the existing knowledge of suitable indications, feasibility, and clinical outcomes of PT. Moreover, different hypofractionated schedules will be collected in order to compare toxicity profiles and clinical outcomes of the two regimes.

## Methods/design

### Participant eligibility criteria

All patients with any type of oncologic disease (benign and malignant disease) who will be eligible for PT (according to international, national and institutional recommendations) and able to provide informed consent (themselves, or through a designated legal guardian) will be included in the present registry. All tumor histologies located in any sites in the body will be considered. No limitation regarding the treatment approach (surgery and/or systemic treatments) will be applied. This wide-range approach takes into account that (1) indications to protons are continuously evolving thanks to the growing literature evidence (2) as the power registry will collect data from different centers, indications to PT could be different among facilities located in different European Countries (3) the power registry allows to collect data from patients enrolled in different clinical trials (if permitted) which could have specific inclusion criteria. Excluded subjects will be pediatric patients (< 18 years old) and patients with mental disorders or impediments of any kind of nature that may prevent proper understanding of informed consent.

Potential registry participants will be recruited during regular clinical activities, multidisciplinary meetings, and outpatient or inpatient visits at all participating centers, or through referral from other centers. Patients enrolled in clinical trials will also be included.

Subject registration procedures will start concomitantly with the beginning of the clinical activity of the IEO Proton Therapy Facility planned for November 2023. Data collection will continue until the end of the follow-up period. The collection is planned for a full 5-year duration. Follow-up will proceed for at least 2 additional years and analyses will be conducted for further 3 years. Therefore, the project has a planned minimum length of 10 years (5 years of data collection, 2 years of follow-up of the last enrolled patient, and a further 3 years of analysis).

### Treatment characteristics

Participants will be treated with the IBA (Ion Beam Applications, Louvain-la-Neuve, Belgium) Proteus®ONE and/or Proteus®PLUS systems according to the best clinical practice following national and international guidelines.

### Registry design

The *POWER* registry will be a prospective data collection repository managed at the European Institute of Oncology (IEO IRCSS, Milan, Italy). Additional Proteus®ONE Centers in Europe may participate in the present registry. After two years, the registry may be extended to other facilities equipped with IBA products other than Proteous®One machine (e.g. Proteus®PLUS Centers). Any protocol modifications (e.g. changes to eligibility criteria or participating centres) will be communicated through published protocol amendments.

The Governance with additional PT facilities will be defined according to scientific collaboration agreements that will be signed between IEO and participating centers.

In the platform, pseudoanonymized data will be collected and stored respecting all the privacy and security criteria, to prevent data leaks. Data integrity, i.e. the overall accuracy, completeness, and consistency of data, as well as safety in regard to regulatory compliance (e.g. General Data Protection Regulation (GDPR) compliance)) [8] and security, will be maintained by carefully defined processes, rules, and standards. Among all the different institutions, e-mail addresses and regular web meetings will be the main communication channels to share updates, issues, and ongoing results.

The platform will integrate both already available and newly implemented data repositories and will be fully integrated with the already existing IEO infrastructure. In this multifunctional platform collected data will include:

- Clinical data
  - patients’ medical history and comorbidities;
  - Quality of life (QoL) assessed with validated international questionnaires, as well as social behavior and psychological distress data with the collaboration of dedicated specialists, will be collected at baseline and subsequently defined timepoints;
  - acute and late toxicity events, collected according to internationally-validated scales, to identify the toxicity profile of PT;
  - costs borne by patients and institutions, logistic issue and information about patients’ discomfort for cost/effectiveness analyses based on real-world data.
- Questionnaires collecting data on patients’ quality of life, as well as toxicity scales, will be defined according to the patients, tumor, and treatment characteristics.
- Imaging data: computed tomography (CT), ultrasound (US), magnetic resonance imaging (MRI), and positron emission tomography (PET) at baseline and at defined time points (simulation, in-room set-up verification during the treatment, follow-up) will be collected.
- Dosimetric data:
  - Dose-volume histogram (DVH) extracted parameters;
  - Constraints used for the optimization process.
- Biological data:
  - tissue specimens, including diagnostic cytologic and pathological specimens (at baseline);
  - patients’ blood and saliva samples (for cancers of the head and neck district) at baseline and defined time points.

Biological samples will be obtained for diagnostic purposes with standardized procedures and will remain stored in the pathological laboratory of each Institution. In the case of the exchange of samples among the participant centers, each step will be defined according to specific agreements between Institutions; samples will be accessible only to the authorized personnel and once the registry is ended they will be disposed of according to Institutional regulations.

**Fig. 1:**
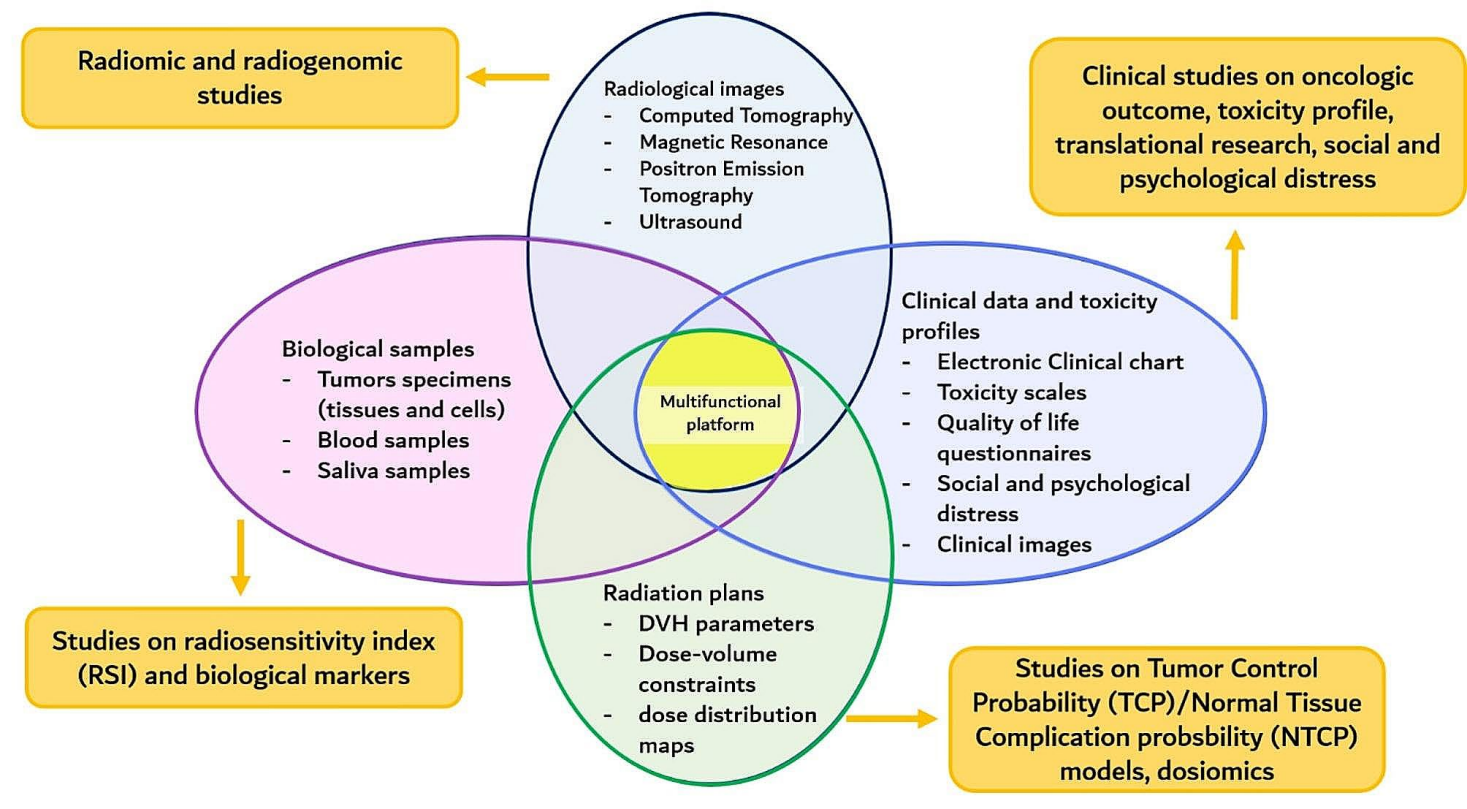
A schematic representation of data collected in the platform

All the data will be collected in a REDCap® (Research Electronic Data Capture) database. REDCap is a secure web platform for building and managing online databases and streamlining processes offering a vast array of tools that can be tailored to virtually any data collection strategy. Moreover, REDCap software has an intuitive user interface, improves data entry through real-time validation rules (e.g. automated data type and range check), and provides easy data manipulation and export to common statistical packages (SPSS, SAS, Stata, R/S-Plus). Figure 1 provides a schematic representation of data collected in the proton registry platform.

### Registry endpoints

The primary aim of the *POWER* registry is to provide a tool for systematic, prospective, harmonized, and multidimensional high-quality data collection to promote knowledge in the field of PT and investigate the use of hypofractionation in PT.

Moreover, data collected in the present registry will be available to support several research fields such as clinical, psychological, cost/effectiveness, radiologic, and pathological studies.

Endpoints and outcome measures will be evaluated in terms of treatment efficacy (primary outcomes) and treatment toxicity (secondary outcomes):

- Primary outcomes:
  1. tumor response rate according to Response Evaluation Criteria in Solid Tumours (RECIST) criteria [9]; other than RECIST, different response evaluation criteria could be used for specific tumors (for example, RANO criteria for tumors of the central nervous system [10]) or in specific clinical settings (for example immune-related immune-responses RECIST (irRECIST)criteria for tumor treated with immunotherapy [11]).
  2. the percentage of tumor recurrence assessed by Progression Free survival (PFS) rate;3) rate of death assessed by overall survival (OS);
- Secondary outcomes:
  1. incidence of acute and late toxicity according to Common Terminology Criteria Adverse Events (CTCAE V 5.0) [12];
  2. patients’ quality of life assessed by internationally validated European Organization for Research and Treatment of Cancer (EORTC) questionnaires;
  3. patients’ pain assessed by Visual Analogue Score;
  4. patients’ quality of life assessed by Quality Adjusted Life Years (QALYs).

Moreover, cost-effectiveness analysis will be carried out by dedicated questionnaires administered to all patients enrolled in the proton registry study.

These objectives will be developed at three different levels as reported in the following details:

### Level 1: clinical research

Clinical data concerning epidemiology, patients’ social status, habits, comorbidity, medical history, treatment characteristics, outcome, and toxicity will be collected for all enrolled patients. These data will be used for different lines of research:

1.1 *Outcomes and toxicity*: clinical data will be collected in order to evaluate clinical outcomes and toxicities.
1.2 *Quality of life*: patients’ quality of life, as well as analysis of ethical, social, and psychological parameters (collected through collaboration with dedicated specialists), will be collected. Collected data will be used to investigate the impact of new treatment approaches on both patients’ and caregivers’ perception according to different parameters such as age, geographical areas, social and familiar environments. Moreover, differences between patients treated with PT and those treated with standard photon-based approach will be collected, analyzed, and reported.
1.3 *Cost/effectiveness analysis*: the cost/benefit ratio of PT administered with different fractionation schedules will be analyzed using different parameters such as the patient’s discomfort and quality of life, logistic issues, and national health system costs. Standardized parameters (e.g. QALYs) will be used to report the obtained results and to compare different approaches.

### Level 2: Radiological and radiobiological research

Radiologic images related to CT, US, MRI, and PET will be collected for all patients at baseline and during the follow-up period according to the daily clinical practice. Moreover, simulation CT, as well as all radiologic images acquired during the radiation treatment, will be collected and analyzed for scientific purposes. In detail, different fields of interest will be developed:

2.1 *Radiomic analysis*: quantitative data retrieved from both radiologic images and PT treatment plans will be analyzed in order to explore radiomic and dosiomic features for the development of new predictive and/or prognostic models.
2.2 *Dosiomic analysis*: dosiomic analysis retrieved from dose distribution calculated by the treatment plans will be analyzed in order to develop prognostic and predictive models of both clinical outcomes and toxicity.
2.3 *Radiobiological modeling*: clinical, radiological, and dosimetric data will be integrated in biological models in order to both validate already published and implement new biological parameters. In particular, Normal tissue complication probability (NTCP) models and Tumor control probability (TCP) models will be used to define toxicity parameters and outcomes endpoint, respectively. The impact of such models according to the different fractionation schedules (hypofractionation vs. standard fractionation) will be analyzed.

### Level 3: Biological and translational research

Biological samples such as cytologic and pathological specimens, blood samples and saliva will be prospectively collected, if not yet available for oncologic procedures, to explore new biological parameters. In particular:

3.1 *Biological biomarkers*: different biological samples will be used for both external validation of already existing biomarkers and identification of new ones will be one of the objectives of the present tumor registry. Specific protocols for sample collection, storage, and analysis will be implemented according to tumor sites, histology, and treatment schedule.
3.2 *Genomic data analysis*: identification of genetic patterns of radiosensitivity and/or radioresistance will be explored on different biological samples. In particular, radiosensitivity index (RSI) [13] will be investigated aiming to achieve a genomic-adjusted radiation dose (GARD) model [14] to be used in clinical practice.

Results of patients treated with PT will be presented as a prospective data collection. Moreover, when possible, results will be compared with: (1) literature data related to comparable cohorts of patients treated with proton- and photon-based approaches; (2) retrospective analysis of comparable cohorts of patients treated with photons at the involved institutions. All the analyses will be supported by the use of advanced statistical analysis.

### Statistical power consideration

Given that this study is a registry and not a study design with a formal hypothesis to be tested, sample size considerations were carried out with a view to exploratory analyses of toxicity as main endpoint in case of hypofractionated regimen.

Limited scientific evidence is currently available regarding the comparison between hypofractionated and standard fractionation schedules in patients treated with protons. Therefore, in order to provide a tentative estimation of sample size for the current registry, results provided by studies performed with photon-based techniques have been considered. Breast and prostate cancer studies have been chosen because both had a large number of analyzed patients and a wide use of hypofractionated schedules in daily clinical practice [15, 16]. Given data published by Whelan et al. [15] regarding toxicity after hypofractionated regimen at 5 years, a sample size of 562 produces a two-sided 95% confidence interval from 1.5 to 4.5% for a proportion of late toxicity of 3%. Given the data from Aluwini et al. [16], regarding toxicity after hypofractionated regimen and frequency at day ≥ 16 urination/voiding, a sample size of 689 produces a two-sided 95% confidence interval from 9.5 to 14.5% when the sample proportion is 12%. If we consider as main endpoint acute toxicity, given the results found in Aluwini et al. [17], a sample size of 1024 produces a two-sided 95% confidence interval from 18 to 23% for a proportion of toxicity 20%. An overall sample size of 2000 patients should allow us to estimate with enough precision acute and late toxicity of main cancer sites.

### Ethical aspects

The registry will be managed according to the Declaration of Helsinki/Tokyo and to Good Clinical Practice guidelines. The protocol was registered at clinicaltrials.gov (NCT05860361) and has been approved by Ethics Committee of the coordinating center IEO e Centro Cardiologico Monzino (n. R1760722-IEO 1885). The protocol will receive Ethics Committee approval of all the participating sites.

The registry will be conducted in compliance with the protocol, Good Clinical Practices (GCP), International Conference on Harmonization (ICH) Guidelines (E6) for GCPs standards as adopted by the Food and Drug Administration (FDA), and associated Federal regulations, and all applicable institutional research requirements (ref. “Investigation of medical devices for human subjects - Good clinical practice (ISO 14155:2020)).

After a complete explanation of the objectives and modalities of the registry, each patient is required to give written informed consent for participation in the registry and for the collection and storage of biological specimens.

Potential benefits to the individual research registry participant include standardized data collection, monitoring, and follow-up processes. Benefits to science consist in the availability of a prospective data collection obtained through a structured database containing high-quality clinical, radiological, and dosimetric data. A potential risk consists in the possible leak of patients’ data. All policies currently available at IEO to preserve the privacy and security of collected data will be applied to the current registry.

### Funding sources

The *POWER* registry project will be funded by IBA (Ion Beam Applications, Louvain-la-Neuve, Belgium) according to a Collaboration Agreement signed by IBA and IEO on 16th September 2021. IBA will not play any role in the registry design, participant selection, as well as in the data management, and the analysis and interpretation of the data. Moreover, other national and international institutional grants and private funding might support the activities related to the project.

## Discussion

It is estimated that approximately one out of two cancer patients should receive RT at least once in the course of their disease in Europe [18]. Within RT techniques, due to the depth-dose properties of proton particles PT has become more and more available with potential benefits in terms of toxicities and tumor control. PT can be considered, in some respects, an established treatment approach, with more than 100 facilities worldwide each treating approximately 400 per year [19]. Nevertheless, widespread discussion regarding the lack of evidence of superior benefits makes PT a technique that still needs to find its role in radiation oncology. Indeed, how much the dose reduction to normal tissues and organs outside the target areas translates into actual clinical benefits compared to the standard photon-based therapy has yet to be demonstrated in some settings. Moreover, major challenges in PT are the cost and space required by the infrastructure, which make facilities still relatively uncommon and lead to the difficulty of building solid evidence and corroborating existing ones.

Moreover, the use of hypofractionated schedules is gaining a growing interest in the setting of patients treated with PT. Preliminary results confirmed the feasibility and efficacy of hypofractionated PT in different clinical settings [2–6]. From a radiobiological point of view, as the dose per fraction increases, there is a decrease in the RBE of protons. This phenomenon is due to the fact that at a higher dose/fraction the cell survival curves of protons and photons are less separated than observed at a lower dose/fraction. Therefore, in the case of hypofractionation, the RBE value of protons is closer to photons than in the case of standard fractionation across the different alfa/beta values [1, 20]. Experimental data presented by Paganetti et al. showed that the average RBE increases with LET from ~ 1.1 in the entrance region, to ~ 1.7 in the distal fall-off in a spread-out Bragg peak for dose/fraction of 2 Gy. These values of RBE decreased by up to 10% for 6 Gy irradiations [21]. From a clinical point of view, the use of hypofractionation presents several advantages: (1) the reduction of the overall treatment time; (2) the reduction of logistic discomfort for the patients; (3) optimization of the still limited available PT resources on either institutional and national level; 3) minimize the impact on the environment through a potential reduction of the carbon footprint. Therefore, one of the main goals of the present Registry is to collect data of patients treated with hypofractionated schedules in order to provide a scientifically relevant contribution in refining both clinical indications and technical aspects of its use in the setting of PT.

The presented registry aims to prospectively collect data from PT treatments across different institutions with the final aim to accumulate robust scientific pieces of evidence regarding indication and clinical results of PT with a particular focus on hypofractionation. The registry will result in a large and robust database able to support the building of scientific solid evidence and provide a useful tool to collect high-quality data among different proton facilities and promote clinical/translational research collaborations. The dataset will also be highly homogeneous, since the collection of all data, including images and biological samples, will be standardized for all centers.

The present registry is in line with other similar initiatives promoted by Scientific Societies. A collaboration between the European Organization for Research and Treatment of Cancer (EORTC) and the European Society for Radiotherapy and Oncology (ESTRO) set up E^2^-RADIatE (EORTC–ESTRO RADiation Infrastructure for Europe; EORTC 1811), a common research platform to collect real-world data of cancer patients treated with RT [22]. The aim is to support RT research and to provide evidence of the role of RT in a multidisciplinary approach, in order to generate robust data in cancer treatment and to further develop and integrate the discipline into therapeutic strategies. E²-RADIatE comprises 3 subcohorts of oligometastatic diseases (OligoCare), patients treated with particle therapy (ParticleCare), and re-irradiations treatments (ReCare).

Particularly, ParticleCare (EORTC 1833-RP) aims to recruit all patients treated with protons and C-ions in European centres to provide an effective data-sharing platform [22, 23]. While OligoCare has already enrolled 1500 patients, enrolment within ParticleCare and ReCare has yet to be started. The primary endpoint of E²-RADIatE is to assess the number of patients treated with RT within 5 years, while secondary aims are disease-free survival, loco-regional control, distant metastasis-free survival, overall survival, and incidence of adverse RT-related events.

Another project currently developing an infrastructure for automatic data registration related to PT is the PROTRAIT (PROton Therapy ReseArch regIsTry) [24] initiative in the Netherlands. The specific aim of the project is to set up an infrastructure for automatic data registration related to novel PT in the Netherlands to validate and strengthen the model-based approach used in this Country.

In order to join forces between RT professionals worldwide to build a high-quality shared dataset of information on PT the platform presented in our project has been built to be also possibly integrated with other already available registry platforms.

Compared to the ongoing projects, the present registry will prospectively collect not only clinical data but also radiologic images and dosimetric data obtained from the treatment plan (Level 2 in the present registry) as well as biological samples (translational research Level 3). Moreover, analyses on the impact of PT on patients’ quality of life, and cost-effectiveness studies will be implemented through the collected data. In addition, subanalyses regarding results of hypofractionated schedules in terms of both oncological outcomes and toxicity profile will provide further scientific evidence aiming to implement this approach in daily clinical practice. This latter represents the primary goal of the present data collection due to either the still limited number of facilities actually in operation or the high costs of the PT which represent among the most important limitations for the widespread implementation of this technique in the setting of cancer patients cure.

We are aware that the scientific level I evidence can be achieved mainly by controlled trials. However, real-world data (such as those provided by tumor registries) have been recognized as a valuable tool aiming to help validate findings from clinical trials and confirm (or not) the effectiveness and safety of some treatments [25]. Therefore, the aim of the present registry is to optimize data collection of patients treated with PT in order to provide reliable real-world data on the topic.

Moreover, subgroup analysis will be conducted in order to provide information on homogeneous cohorts of patients and limit the spread of the collected data.

Finally, as the planned length of the project could be not sufficient to address specific issues (like long-term toxicity in surviving patients) an extension of the patients’ enrollment and follow-up will be evaluated.

## Conclusion

The importance of generating high-quality data for PT is paramount for building solid scientific evidence and individuating patients who could indeed benefit from PT. In this scenario, the present multicentric and multi-source prospective registry may substantially foster the research endeavor at leading cancer research institutions worldwide.

## Data Availability

Not aspplicable.

## References

[1] Santos A, Penfold S, Gorayski P, Le eH. «The Role of Hypofractionation in Proton Therapy», *Cancers*, vol. 14, fasc. 9, Art. fasc. 9, gen. 2022, 10.3390/cancers14092271. 10.3390/cancers14092271 PMC9104796 35565400

[2] Alterio D et al. apr., «Hypofractionated proton therapy in breast cancer: where are we? A critical review of the literature», *Breast Cancer Res. Treat*, vol. 192, fasc. 2, pp. 249–263, 2022, 10.1007/s10549-022-06516-4. 10.1007/s10549-022-06516-4 35025004

[3] Hoppe BS, et al. «Chemoradiation with Hypofractionated Proton Therapy in Stage II-III Non-small Cell Lung Cancer: a YYY phase 1/2 Trial». Int J Radiat Oncol. mar. 2022;0360301622002383:S. 10.1016/j.ijrobp.2022.03.005. 10.1016/j.ijrobp.2022.03.005 35306151

[4] Volpe S, et al. «Hypofractionated proton therapy for non-small cell lung cancer: ready for prime time? A systematic review and meta-analysis». Cancer Treat Rev. nov. 2022;110:102464. 10.1016/j.ctrv.2022.102464. 10.1016/j.ctrv.2022.102464 36194908

[5] Nakajima K et al. «Patient-Reported Quality of Life Outcomes after Moderately Hypofractionated and Normofractionated Proton Therapy for Localized Prostate Cancer», *Cancers*, vol. 14, fasc. 3, p. 517, gen. 2022, 10.3390/cancers14030517. 10.3390/cancers14030517 PMC8833499 35158785

[6] Parzen JS et al. «Hypofractionated proton beam radiotherapy in patients with unresectable liver tumors: multi-institutional prospective results from the Proton Collaborative Group», *Radiat. Oncol*, vol. 15, fasc. 1, p. 255, dic. 2020, 10.1186/s13014-020-01703-3. 10.1186/s13014-020-01703-3 PMC7643436 33148296

[7] Poon DMC, Wu S, Ho L, Cheung KY, Yu eB. «Proton Therapy for Prostate Cancer: Challenges and Opportunities», *Cancers*, vol. 14, fasc. 4, p. 925, feb. 2022, 10.3390/cancers14040925. 10.3390/cancers14040925 PMC8870339 35205673

[8] 14:00–17:00, «ISO 14155:2020», ISO. Consultato: 2 febbraio 2023. [Online]. Disponibile su: https://www.iso.org/standard/71690.html.

[9] Eisenhauer EA et al. «New response evaluation criteria in solid tumours: Revised RECIST guideline (version 1.1)», *Eur. J. Cancer*, vol. 45, fasc. 2, pp. 228–247, gen. 2009, 10.1016/j.ejca.2008.10.026. 10.1016/j.ejca.2008.10.026 19097774

[10] Wen PY et al. apr., «Updated Response Assessment Criteria for High-Grade Gliomas: Response Assessment in Neuro-Oncology Working Group», *J. Clin. Oncol*, vol. 28, fasc. 11, pp. 1963–1972, 2010, 10.1200/JCO.2009.26.3541. 10.1200/JCO.2009.26.3541 20231676

[11] Seymour L et al. mar., «iRECIST: guidelines for response criteria for use in trials testing immunotherapeutics», *Lancet Oncol*, vol. 18, fasc. 3, pp. e143–e152, 2017, 10.1016/S1470-2045(17)30074-8. 10.1016/S1470-2045(17)30074-8 PMC5648544 28271869

[12] National Cancer Institute, «Common Terminology Criteria for Adverse Events», in Definitions, Qeios., 2020. 10.32388/ERJXIQ.

[13] Eschrich SA et al. «A Gene Expression Model of Intrinsic Tumor Radiosensitivity: Prediction of Response and Prognosis After Chemoradiation», *Int. J. Radiat. Oncol*, vol. 75, fasc. 2, pp. 489–496, ott. 2009, 10.1016/j.ijrobp.2009.06.014. 10.1016/j.ijrobp.2009.06.014 PMC3038688 19735873

[14] Scott JG et al. feb., «A genome-based model for adjusting radiotherapy dose (GARD): a retrospective, cohort-based study», *Lancet Oncol*, vol. 18, fasc. 2, pp. 202–211, 2017, 10.1016/S1470-2045(16)30648-9. 10.1016/S1470-2045(16)30648-9 PMC7771305 27993569

[15] Whelan TJ et al. feb., «Long-Term Results of Hypofractionated Radiation Therapy for Breast Cancer», *N. Engl. J. Med*, vol. 362, fasc. 6, pp. 513–520, 2010, 10.1056/NEJMoa0906260. 10.1056/NEJMoa0906260 20147717

[16] Aluwini S et al. apr., «Hypofractionated versus conventionally fractionated radiotherapy for patients with prostate cancer (HYPRO): late toxicity results from a randomised, non-inferiority, phase 3 trial», *Lancet Oncol*, vol. 17, fasc. 4, pp. 464–474, 2016, 10.1016/S1470-2045(15)00567-7. 10.1016/S1470-2045(15)00567-7 26968359

[17] Aluwini S et al. mar., «Hypofractionated versus conventionally fractionated radiotherapy for patients with prostate cancer (HYPRO): acute toxicity results from a randomised non-inferiority phase 3 trial», *Lancet Oncol*, vol. 16, fasc. 3, pp. 274–283, 2015, 10.1016/S1470-2045(14)70482-6. 10.1016/S1470-2045(14)70482-6 25656287

[18] Delaney G, Jacob S, Featherstone C, Barton eM. «The role of radiotherapy in cancer treatment: Estimating optimal utilization from a review of evidence-based clinical guidelines», *Cancer*, vol. 104, fasc. 6, pp. 1129–1137, set. 2005, 10.1002/cncr.21324. 10.1002/cncr.21324 16080176

[19] «PTCOG - Facilities in Operation». Consultato: 3 gennaio 2023. [Online]. Disponibile su: https://www.ptcog.ch/index.php/facilities-in-operation-restricted.

[20] Friedrich T. «Proton RBE dependence on dose in the setting of hypofractionation», *Br. J. Radiol*, vol. 93, fasc. 1107, p. 20190291, mar. 2020, 10.1259/bjr.20190291. 10.1259/bjr.20190291 PMC7066967 31437004

[21] Paganetti H. «Relative biological effectiveness (RBE) values for proton beam therapy. Variations as a function of biological endpoint, dose, and linear energy transfer», *Phys. Med. Biol*, vol. 59, fasc. 22, pp. R419-472, nov. 2014, 10.1088/0031-9155/59/22/R419. 10.1088/0031-9155/59/22/R419 25361443

[22] European Organisation for Research and Treatment of Cancer - EORTC, «E2-RADIatE: EORTC-ESTRO RADiotherapy InfrAstrucTure for Europe», clinicaltrials.gov, Clinical trial registration NCT03818503., nov. 2019. Consultato: 16 gennaio 2023. [Online]. Disponibile su: https://clinicaltrials.gov/ct2/show/NCT03818503.

[23] Frédéric «Cohorts», E2-RADIatE. Consultato: 17 gennaio 2023. [Online]. Disponibile su: https://project.eortc.org/e2-radiate/cohorts/.

[24] «Use case.: PROTRAIT, national registration of proton therapy data| The Personal Health Train». Consultato: 2 febbraio 2023. [Online]. Disponibile su: https://pht.health-ri.nl/use-cases/health-research/use-case-protrait-national-registration-proton-therapy-data.

[25] Botticelli A et al. nov., «Real-World Outcomes of Trastuzumab Deruxtecan in Patients With HER2 + Metastatic Breast Cancer: The DE-REAL Study», *The Oncologist*, p. oyad308, 2023, 10.1093/oncolo/oyad308. 10.1093/oncolo/oyad308 PMC10994255 37995313

